# Measuring spatial co-occurrences of species potentially involved in *Leishmania* transmission cycles through a predictive and fieldwork approach

**DOI:** 10.1038/s41598-021-85763-9

**Published:** 2021-03-24

**Authors:** Marla López, Diana Erazo, Juliana Hoyos, Cielo León, Patricia Fuya, Ligia Lugo, Juan Manuel Cordovez, Camila González

**Affiliations:** 1 Departamento de Ciencias Biológicas, Facultad de Ciencias, Centro de Investigaciones en Microbiología y Parasitología Tropical (CIMPAT), Universidad de Los Andes, Bogotá, Colombia; 2 Grupo de Investigación en Biología Matemática y Computacional (BIOMAC), Departamento de Ingeniería Biomedica, Facultad de Ingeniería, Universidad de Los Andes, Bogotá, Colombia; 3 Laboratorio de Entomología, Instituto Nacional de Salud (INS), Bogotá, Colombia

**Keywords:** Ecological epidemiology, Ecological modelling

## Abstract

The Leishmaniases are a group of neglected tropical diseases caused by different species of the protozoan parasite *Leishmania*, transmitted to its mammalian hosts by the bites of several species of female Phlebotominae sand flies. Many factors have contributed to shifts in the disease distribution and eco epidemiological outcomes, resulting in the emergence of Cutaneous Leishmaniasis outbreaks and the incrimination of vectors in unreported regions. New research development is vital for establishing the new paradigms of the present transmission cycles, hoping to facilitate new control strategies to reduce parasite transmission. Hereafter, this work aims to model and infer the current transmission cycles of Cutaneous Leishmaniasis in Colombia defined by vector and mammal species distributed and interacting in the different regions and validate them by performing sand fly and mammal collections. Vector-host co-occurrences were computed considering five ecoregions of the Colombian territory defined by the World Wide Fund for Nature (WWF) and downloaded from The Nature Conservancy TNC Maps website. Four validation sites were selected based on Cutaneous Leishmaniasis prevalence reports. Sand flies and mammals captured in the field were processed, and species were defined using conventional taxonomic guidelines. Detection of infection by *Leishmania* was performed to identify transmission cycles in the selected areas. This study uses predictive models based on available information from international gazetteers and fieldwork to confirm sand fly and mammalian species' sustaining *Leishmania* transmission cycles. Our results show an uneven distribution of mammal samples in Colombia, possibly due to sampling bias, since only two departments contributed 50% of the available samples. Bats were the vertebrates with the highest score values, suggesting substantial spatial overlap with sand flies than the rest of the vertebrates evaluated. Fieldwork allowed identifying three circulating *Leishmania* species, isolated from three sand fly species. In the Montane Forest ecosystem, one small marsupial, *Gracilinanus marica*, was found infected with *Leishmania panamensis*, constituting the first record of this species infected with *Leishmania*. In the same locality, an infected sand fly, *Pintomyia pia,* was found. The overall results could support the understanding of the current transmission cycles of Leishmaniasis in Colombia.

## Introduction

Phlebotomine sand flies are the only known natural vector of the protozoan parasite *Leishmania*, the causative agent of Leishmaniasis^1^. The disease is classified as a zoonosis, and the parasite transmission cycle is supported by hematophagous female sand fly vectors and mammalian reservoirs^2^. Humans are considered incidental hosts, affected when bitten by a sand fly carrying the parasite. Sand flies get infected when feeding upon certain mammalian species defined as reservoirs of the disease^3^. Depending on the clinical symptoms and the parasite species involved in the infection, three forms of the disease have been described: cutaneous, visceral, and mucocutaneous leishmaniasis^4^. The World Health Organization has classified Colombia as an endemic country, presenting all the clinical forms of the disease^5^. Of the 110.366 cases reported in Colombia from 2008 to 2018, 98.5% belong to the cutaneous (CL) form of the disease, consisting of dermatologic lesions causing disability if not well treated^4,6,7^. Mucocutaneous leishmaniasis (MCL) is a subsequent reaction to untreated cutaneous lesions and may affect nasal cavities, the septum, and the palate^4,8^; this form accounts for 1.4% of the cases recorded^8^. Visceral leishmaniasis (VL) is the only form with a fatality rate above 90% in the absence of treatment; it is characterized by liver and spleen swelling and severe anemia^8^. In Colombia, the disease occurs in almost every department except for the islands of San Andres and Providencia, where vectors have not been reported^6,7,9^. Nine *Leishmania* species are known to co-occur in the Country with *Leishmania braziliensis* and *Leishmania panamensis* being widely distributed and responsible for the highest number of human infections^10^. Regarding the visceral form of the disease, *Leishmania infantum* has been identified as the causative agent^9,11–13^.

Regarding vector species, the subfamily Phlebotomine gathers approximately 988 species of sand flies distributed around the world^14,15^. Neotropical countries have the highest number of reported species, and Colombia is the second with 163 identified species^14,15^. Of these, 14 are proven vectors of CL, but due to the ecological complexity and biodiversity present in the country, other species are suspected of playing a role as vectors^9,15^. Sand fly distribution ranges have been modified due to ecosystem transformation, and their association to conserved ecosystems is no longer restricting the disease to sylvatic areas^9,11^. Many factors have contributed to shifts in the disease’s spatial distribution and changes in eco-epidemiological outcomes have been reported^16^. Which have resulted in the notification of CL outbreaks in new municipalities and the incrimination of vectors in unreported regions, directly correlated with increases in the incidence of the disease in the country^14^. Human migration has also promoted Leishmaniasis spreading, especially because new human settlements in unexploited ecosystems enable the connection between the sylvatic cycle and the new peridomestic and domestic environments^2,16^. Variation in Leishmaniasis’ transmission cycles is of concern, mainly because new vector-parasite associations have been found. There is evidence of sylvatic vectors’ remarkable ability to adapt to transformed ecosystems and feed on alternative blood meal sources such as humans and domestic animals^16^. Even though reservoirs play a crucial role in disease dissemination, little information regarding their epidemiological role is available since most studies are restricted to entomological surveillance in urban settings. Description of *Leishmania* reservoirs is then scarce and mainly based on reports of natural infection, limiting the understanding of potential vector-reservoir interactions^16^. This complex set of species’ interactions imposes strong challenges in outbreak prevention and control interventions^2,16^, thus simultaneous evaluations of vector and reservoir species spatially co-occurring are of great relevance.

Different approaches have been used to infer the species potentially involved in *Leishmania* transmission cycles. Ecological niche modeling has provided a tool to infer species that could potentially be distributed in transmission areas^9,17^, while the use of biotic interaction networks has been proposed as a tool to determine the potential of mammalian species to interact with sand fly vectors, based on co-occurrence analysis^18^.

From this perspective, the objective of this work was to evaluate the patterns of co-occurrence between vector and mammal species in Colombia and to infer the species potentially involved in *Leishmania* transmission cycles. An initial predictive approach was implemented to define mammal-sand fly co-occurrences in five ecoregions, and to define areas of potential sand fly presence based on the score function by Stephens et al.)^18^. Fieldwork was carried-out to confirm the presence of predicted sand fly species as well as to detect infection by *Leishmania* in mammals and sand flies captured. The results obtained aim to offer new insights in order to tackle the disease complexity and, therefore, aid in the delimitation of risk areas where implementation of prevention strategies should be conducted.

## Results

### Measuring vector-mammal co-occurrences in a geographic space

From the revised biodiversity databases, 8559 single mammal records were obtained, including 173 genera and 410 species. The departments with the highest number of records were Antioquia (n = 2599) and Meta (n = 2155), contributing more than 50% of the collection records. Those with the least amount of records were Chocó and Quindío with two records, followed by Arauca (n = 4) and Guainía (n = 5). The mammal species with the highest number of occurrences were *Proechimys oconnelli* (n = 1110), *Didelphis marsupialis* (n = 511), and *Proechimys cayennensis* (n = 280). A total of 251 species had less than 10 records, and 78 had individual records, due to sampling bias across the national territory.

The sand fly species database included 687 unique records of 20 species of sand flies with medical importance in the transmission of Leishmaniases in Colombia. The species with the highest number of occurrences were *Lutzomyia gomezi* (n = 97), *Lutzomyia longipalpis* (n = 55), *Nyssomyia yuilli* (n = 53) and *Psathyromyia shannoni* (n = 52). On the contrary, *Psychodopygus carrerai thula* (n = 5), and *Pintomyia youngi* (n = 10) were the species with the least occurrences.

Regarding species co-occurrences, the Montane forest ecoregion grouped the largest number of vectors (17 species) and mammals (12 orders, 124 genera), followed by the Dry (mammals 10 orders, 113 genera; vectors 12 species) and Moist forests (mammals 10 orders, 112 genera; vectors 15 species). Vector species in these regions showed numerous associations with various mammalian species, which may be the result of high species richness.

Regarding heatmap analysis at the level of Family, in general, Chiroptera (particularly Phyllostomidae), Didelphimorphia and Rodentia had high score values with *B. flaviscutellata*, *L. gomezi*, *N. antunesi*, *N. yuilli* and *N. panamensis*, while *P. shannoni* is more often found co-occurring with rodents, as does *P. columbiana*. In Moist forest, the dominant vector species were *Nyssomyia yuilli*, *Nyssomyia antunesi*, and *Psychodopygus panamensis* showing strong scores with species of the Phyllostomidae family. *Carollia* sp., *Glossophaga* sp., *Platyrrhinus* sp. and *Phyllostomus* sp. showed the highest scores for these vector species. Didelphidae and Cricetidae families showed high scores in the Moist forest as well, particularly, *D. marsupialis*, *Philander opossum*, and *Metachirus nudicaudatus* from Didelphidae and *Nectomys* sp. from Cricetidae. Interestingly in this ecoregion *Lutzomyia gomezi* is strongly predicted to interact with bats from Emballonuridae and Phyllostomidae families (Fig. S1).

On the other hand, Lowlands and Xeric shrublands included the lowest number of both vector and mammalian species. These cases resulted in high score values between sand flies and their potential reservoirs, implying high spatial co-occurrences (Fig. S1). In the Lowlands, two sand fly species, *N. antunesi* and *Ps. shannoni* were predicted interacting with the Phyllostomidae bats *Glossophaga longirostris* and *Carollia perspicillata*. Results suggest that *N. antunesi* is the sand fly species that co-occurs with more mammal species in the Lowlands region. In Xeric shrublands, *Lutzomyia gomezi* and *P. evansi* have high score values with diverse mammal species, bats predominantly. At the same time, *Psychodopygus panamensis* and *L. longipalpis* were found co-occurring less with the Chiroptera order. Additionally, the sloths *Bradypus variegatus* and *Choloepus hoffmani* showed high score values with both vector species.

Sand fly species with the largest known distributions, such as *Lutzomyia gomezi* and *Psychodopygus panamensis*^10^, had the highest number of co-occurrences with different species in multiple ecoregions; nonetheless, some of them with low score values. On the contrary, sand fly species from the Verrucarum group (Genus *Pintomyia)* presented a low number of co-occurrences with multiple mammalian species, possibly because these species have a distribution restricted to the Andean region (Montane forest). *Nyssomyia yuilli*, present in four of the five ecoregions, showed co-occurrence with several mammalian species.

**Figure 1:**
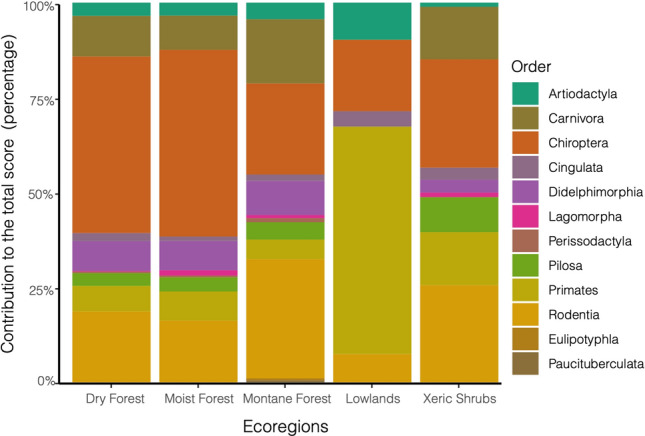
Scores by order per ecoregion. Twelve mammal orders were found in the database records for the five ecoregions that comprise Colombia. The *x-axis* shows the ecoregions. The *y-axis* represents the contribution of each mammal taxonomic order to the total score calculated in each ecoregion (see Supplementary Tables for more detail). Chiroptera presents the highest contribution to the Moist and Dry forests, with more than 50% of the total score in both habitats, followed by Rodentia. Similarly, in the Montane forest and Xeric shrublands, the highest scores are shared between Rodentia and Chiroptera order. Primates represents the highest contribution in the Lowlands and an important contribution in the Xeric shrublands.

Regarding mammals, in all regions except in the Lowlands, Chiroptera showed the highest co-occurrences with sand fly species, followed by Rodentia and Carnivora orders (Fig. S1). Among regions, the Lowlands differs significantly from the others, where Primates contribute more than 50% to the total score, and Artiodactyla seems to play an important role as well. Pilosa and Didelphimorphia co-occurrences with sand flies are relevant, being nearly 12.5% in all regions except in the Lowlands (Fig. 1).

Regarding the score map by ecoregion, sampling sites varied with Dry ecoregion showing the highest score (33.08), followed by Montane (29.76), Moist (13.52) and Lowlands (3.13) (Fig. 3).

### Fieldwork co-occurrence consensus

The correct classification index (CCI) was in general low for the four field sites (closer to 0 that to 1) being the site in the Lowlands the one with higher number of shared species (CCI = 0.33), followed by the montane 0.18, moist 0.17, and Dry 0.12. In the following sections, collected sand fly and mammal species, as well as their infection status are described.

### Specimen collection and identification

In total, 2336 Phlebotomine sand flies were captured (1064♂; 1272♀), and 1641 were identified to species (Table 1). The site with the largest number of captures was Salitral, and the one with the least amount was Cafreria (Table 1). In the four collection sites, 73 mammalian individuals were captured. Tissue and blood samples were taken from 68 of them, which were posteriorly released. The remaining five, captured in El Eden and Cafreria, were euthanized (Table 2).

**Table 1:** Phlebotomine sand fly species identified in each collection site showing number of individuals collected.

Municipality (collection site)		Sahagun (Salitral)		Icononzo (Cafreria)		Nilo (El Eden)		Tauramena (Los potrillos)		Total
Species identified										
Genus	Species	♂	♀	♂	♀	♂	♀	♂	♀	
*Pintomyia*	*evansi*	297	182							479
*Psychodopygus*	*panamensis*	31	64		2			29	367	493
*Lutzomyia*	*gomezi*	41	38					2	2	83
*Micropigomyia*	*cayennensis*	22	22			100	27			171
*Evandromyia*	*dubitans*	16	5	2		1				24
*Micropigomyia*	*trinidadensis*	3	6	14	37	48	42			150
*Micropigomyia*	*carpenteri*	4	0			7				11
*Pifanomyia*	*rangeliana*	1	1							2
*Brumptomyia*	sp.			53	32					85
*Lutzomyia*	*longipalpis*				4	61	37			102
*Pintomyia*	*pia*				3					3
*Nyssomyia*	*antunesi*							17	0	17
*Lutzomyia*	*torvida*				1					1
*Pressatia*	*choti*							12	8	20
Total		415	318	69	79	217	106	60	377	1641

**Table 2:** Mammal species and number of individuals collected in each locality.

Ecoregion		Montane forest	Dry forest	Lowlands	Total
Department		Tolima	Cundinamarca	Casanare	
Municipality (collection site)		Icononzo (Cafreria)	Nilo (El Eden)	Tauramena (Los potrillos)	
Order	Species				
Chiroptera*	*Carollia perspicillata*			5	5
	*Myotis riparius*			2	2
	*Plathyrrinus brachicephalus*			3	3
	*Artibeus planirostris*			1	1
	*Phyllostomus discolor*			1	1
	*Lophostoma brasiliense*			1	1
	*Chiroptera* sp.			15	15
Didelphimorphia	*Gracilinanus marica*	1			1
	*Marmosa robinsoni*	1			1
	*Marmosops fuscatus*		1		1
	*Didelphidae* sp.			4	4
Rodentia	*Rhipidomys latimanus*	1			1
	*Heteromys anomalus*		1		1
	*Zygodontomys brevicauda*			20	20
	*Rodentia* sp.			16	16
Total		3	2	68	73

*Sampled only in Lowlands.

From the 73 individuals 38 were rodents, 7 marsupials, and 28 bats. The site with the highest capture success was Los Potrillos, also the only location where bats were sampled. For 38 mammal species, validation of species identity through barcode was achieved.

### *Leishmania* infection

Of the 88 sand fly pools (289 female sand flies) processed for *Leishmania* infection, five pools were detected as positive. Infection was confirmed by Sanger sequencing, performed in the DNA sequencing center of Universidad de los Andes. Two pools of *Pintomyia pia* were found infected with *Leishmania panamensis*, in Cafreria from two different traps. Two pools corresponding to *Pintomyia evansi* from Salitral were positive for *Leishmania infantum*, which is the causative agent of visceral leishmaniasis, and one pool of *Psychodopygus panamensis* from Los Potrillos was positive for *Leishmania amazonensis.* Only species with a 100% hit in BLAST were accepted and confirmed as positives.

Of the total samples of mammals processed for *Leishmania* parasite infection, only one individual was positive. The species *Gracilinanus marica*, commonly known as the Northern gracile opossum, was captured in Cafreria and was found infected with *Leishmania panamensis*. The parasite species was confirmed by amplification with both HSP70, ITS, and Cytb primers. To assure the detection was correct, another PCR was performed with primers targeted to KDNA and 18S^19,20^. PCR templates obtained for Cytb and HSP70 were sequenced, and a 100% BLAST hit allowed the final confirmation.

### Species identity confirmation

PCR templates obtained with the COI targeted primers from mammalian samples were sequenced and the species identity was confirmed with Blast. Table S1 describes the mammalian species identified, the identity percentage and collection site. In contrast, Table S2 summarizes the sand fly species that could be identified in each site with their corresponding identity percentage and accession number from NCBI.

Accurate barcode analysis in Phlebotomine sand fly species was limited; therefore, a more specific analysis was performed with the sequences for confirmation. A histogram for the visualization of inter and intraspecific genetic distances was designed (Fig. S2). Genetic distances obtained from K2P distance analysis for intraspecies values ranged between 0 and 4.11% (average intraspecific divergence = 1.58%; Standard deviation = 1.62%) and values between species ranged between 11.80 and 21% (average interspecies divergence = 17.13%; Standard deviation = 2.50%). The values obtained are similar to the results obtained by other authors regarding sand fly barcoding. To confirm the accuracy of the obtained sequences to delimit species identity, the “barcoding gap” was used. The obtained results determine that there is no overlap between the intraspecific and interspecific values and therefore genetic differences between the species is indicated (see Fig. S2)^20,21^. Figure S3 corresponds to the dendrogram based in K2P neighbor joining distances. Reference sequences from each of the identified species were downloaded from GenBank to confirm adequate clustering.

In the dendrogram (Fig. S3), the identified sand fly species formed monophyletic groups with reference sequences, which is confirmed by the genetic divergence values. Only one of the sequences, corresponding to code 242, identified as *Psychodopygus panamensis* could not be confirmed using Blast.

## Discussion

Among vector-borne diseases, Leishmaniases remain predominantly zoonotic, strongly dependent on species present in sylvatic cycles, making surveillance of these species an essential tool to detect circulating parasite species, and anticipating human cases. In this context, the implementation of methodologies that allow the prediction of species potentially involved in sylvatic cycles in a given locality is of great relevance^22^. Using computational methods to infer vector-host co-occurrence from collection data is a known practice in disease ecology that could be applied to several vector-borne diseases, particularly to those where comprehensive collection data on potential vector and hosts species is available. For instance, using this methodology, Ibarra-Cerdeña et al*.* identified synanthropic mammals as important hosts for *Trypanosoma cruzi* transmission in Mexico^23^. Here, we used existing collection records of sand flies and mammals in Colombia, to evaluate their co-distribution, infer species potentially involved in *Leishmania* transmission, and assess the relative risk in five regions with different ecological characteristics.

From the co-occurrence analysis, several mammal species should be regarded as potential reservoirs for *Leishmania*, and special attention should be paid to synanthropic species such as rodents, marsupials and bats, with potential to interact with human populations. The rodents *H. anomalus* in the Dry forest and *Z. brevicauda* in the Lowlands had high score values. Previous studies have shown that although these two species might be useful for *L. evansi* feeding, they are not for *Leishmania infantum* transmission in the Moist forest^24^. Our results suggest that the role of *H. anomalus* and *Z. brevicauda* remains to be determined, since they are abundant^25^ and could interact with other sand fly and *Leishmania* species. Other rodents such as *Proechimys* sp. and *Coendou* sp. were not captured in our field sites but have been suggested as important parasite hosts^15^. Strong co-occurrence values with opossum species were also found, supporting previous findings on *Didelphis marsupialis* role as a potential reservoir in Colombia and a crucial host in Brazil and French Guyana^26^. *Marmosa cinerea, Marmosa* sp*., Marmosops incanus,* and *Gracilinanus agilis*, have been incriminated as hosts in Brazil and found infected with the species *Leishmania amazonensis, L. *(*Viannia*) sp.*, Leishmania braziliensis* and *Leismania guyanensis,* respectively^16^. This data is relevant, considering that three species of opossums were found in both co-occurrence predictions and fieldwork: *Marmosops* sp. in the Dry forest, *Marmosa robinsoni* and *G. marica* in the Montane forest. Interestingly, *Gracilinanus marica* was found infected with *Leishmania panamensis* in the Montane forest site*,* constituting the first record for this species. This opossum is commonly known as Northern Gracile Mouse opossum, and it is distributed in Venezuela and Colombia. It is described as highly tolerant to habitat modification and found in secondary forests^27^.

Several mammalian species presenting the highest score values corresponded to the Chiroptera order; however, the role of bats as reservoirs has not yet been fully defined. In Colombia there are so far no records of infected specimens with *Leishmania,* but some species of bats have been identified as potential reservoirs by various studies in Mexico, Brazil and Venezuela^28–30^. Our results suggest that Phyllostomidae species could play an important role, especially *C. perspicillata* and its potential interaction with *N. antunesi*. Moreover, *C. perspicillata* has been suggested as a potential feeding source for *L. longipalpis*^31^ in Venezuela and reservoir of visceral Leishmaniasis in Brazil^32^.

Regarding collected sand flies, their abundances and species identified in the sites were consistent with the literature and yet, for the first time, *Pintomyia pia* is reported as infected with *Leishmania panamensis*. Even though it has been noted for its anthropophilic habits, this species has not been incriminated as a vector yet^20^. While more research is required before defining a species as a natural vector^33^; this finding proposes a new sand fly-parasite interaction, particularly when considering that it has been identified as the most abundant species in field captures in the Colombian coffee zone^20^.

*Psychodopygus panamensis* was found infected with *Leishmania amazonensis* in the Lowlands ecoregion. Even though *P. panamensis* is a confirmed vector for the transmission of cutaneous leishmaniasis in the country, it is principally known for transmitting *Leishmania panamensis*^34^. However, *P. panamensis* is an abundant vector with a wide distribution and *L. amazonensis* is known for its distribution in this area of the country, therefore the association is not surprising^15,20^.

The modeling approach used in our study did not allow to discriminate the risk of infection in the different ecoregions, since in spite of the varying predicted score values in the four sampling sites, *Leishmania* was found in three of them. Possibly this an outcome of sampling bias in the historic collections, where areas with apparently low predicted sand fly-mammal co-occurrences, could appear as a result of missing information^35^ The low correct classification index in the sampling sites also reflect information gaps, therefore, field collections should increase to better understand sylvatic transmission cycles. In particular, future efforts should focus on those regions where the information is the scarcest, such as the departments located in the Lowlands ecoregion, as shown by the score map.

As expected, infection rates were low, with only one of the 73 captured mammals positive for *L. panamensis*, and five out of 88 sand fly pools positive for *L. panamensis, L. infantum* and *L. amazonensis*, reflecting the low infection rates at which *Leishmania* parasites circulate in sylvatic cycles. Nonetheless, our results provide useful information on the *Leishmania* species present in the different ecoregions, and the potential vectors and mammals involved in transmission cycles.

## Conclusions

The study of host-vector co-occurrences is a valuable theoretical tool for assessing the role of potentially interacting species, particularly when data on species distributions is available. Nonetheless, information gaps make it difficult to perform accurate predictions so fieldwork is required for a deeper understanding of transmission systems. The absence of sand fly species in the database that were found in the field, and the validation of seven sand fly—mammal co-occurrences suggest high underreport and the need of more field studies on Leishmaniasis in Colombia. Sampling bias also impacted the predicted power of the score metric, since score values didn’t correlate with infection; *Leishmania* was detected in predicted high and low risk areas for sand fly-mammal co-occurrences.

From the fieldwork conducted in four ecoregions in Colombia, three were positive for *Leishmania* parasites detected in vector species. Lastly, *Leishmania panamensis* was found in an opossum (*Gracilinanus marica*) from the montane forest site constituting the first record of this species infected with *Leishmania.*

## Methods

### Measuring vector-mammal co-occurrences in a geographic space

To model vector-mammal co-occurrences, two databases, including collection points from mammalian and Phlebotomine sand fly species occurring in Colombia, were assembled. Mammalian species records were obtained from the Global Biodiversity Information Facility free access electronic database (GBIF) [http://www.gbif.org/] and complemented with data published in the national open access data portal “Sistema de Información sobre Biodiversidad de Colombia” (SIB) [http://datos.biodiversidad.co/]. Records from this database were used at the taxonomic level of Order. A published vector species database including Phlebotomine sand fly species of medical importance for the transmission of leishmaniasis in the country was used^9^. Both databases were depurated to ensure data quality: taxonomy was revised, and records with incorrect geographic coordinates were discarded; only unique records were included.

**Figure 2:**
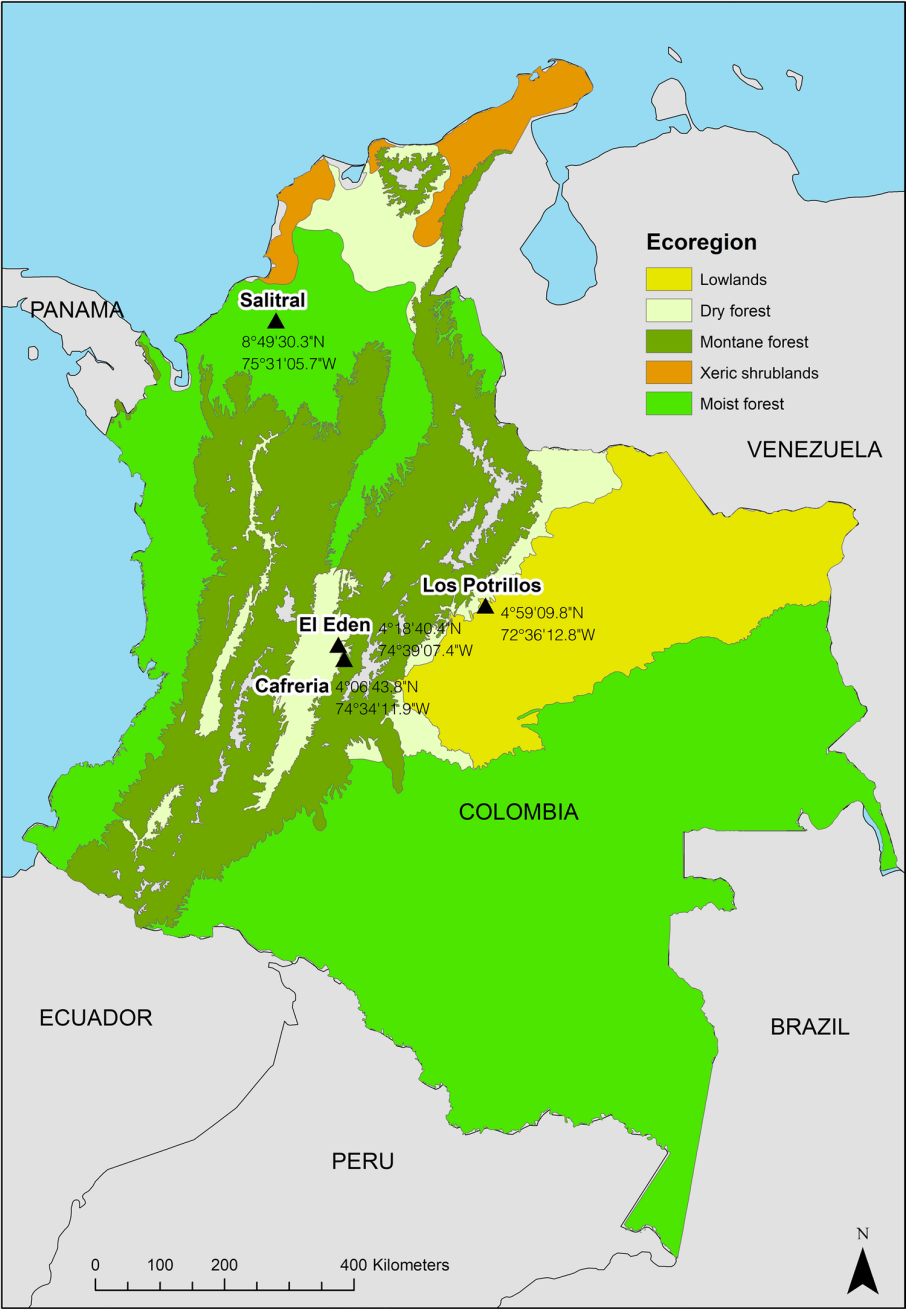
(**A**–**E**) Heat maps and hierarchical clustering (Bray–Curtis) of each ecoregion based on the scores calculated for each co-occurrence event. The color key for the heatmap is shown in the left superior corner, indicating the range of score values. The heatmaps were performed for each ecoregion, and distribution records for sand flies and mammals are shown (**A**) Dry forest, (**B**) Moist forest, (**C**) Montane forest, (**D**) Lowlands, (**E**) Xeric shrublands. Ecoregions in Colombia and study site. This study considered five ecoregions in Colombia for computing sand fly and mammal species co-occurrences: Lowlands, Dry forest, Montane forest, Xeric shrubs and Moist Forest. In 4 out of the 5 ecoregions, fieldwork was conducted to capture sand fly and mammal species: (1) Los Potrillos, Tauramena municipality, department of Casanare, is the collection site selected in the Lowlands ecoregion. (2) Salitral, the Moist Forest collection site, is located in the Municipality of Sahagun in the Cordoba department. (3) Cafreria located in the Icononzo municipality, department of Tolima, was selected within the Dry forest ecoregion. (4) El Eden, Nilo municipality, Cundinamarca department is found in the Montane forest. Field sites coordinates are shown in the map.

Since transmission cycles in *Leishmania* are influenced by local fauna and environmental conditions, we restricted the amplitude of the co-occurrences to specific regions using the Terrestrial Ecoregions, defined by the World Wildlife Fund for Nature (WWF) [Available at: http://maps.tnc.org/gis_data.html], using ArcGis 10.4.1 [ESRI 2016. ArcGIS Desktop: Release 10.4.1. Redlands, CA: Environmental Systems Research Institute.] Five principal Ecoregions were selected: montane forest, moist forest, dry forest, lowlands, and xeric shrublands (Fig. 2).

To construct a predictive model for co-occurences, the probability of finding one taxon, given another one $P(B_{i} |B_{k} )$ was calculated, as a measure of statistical association between the two taxa (Stephens et al. 2009). The score function was used as a proxy, since it is a measure of pair-wise dependencies between taxa $s \left( {\frac{Bi}{{B^{\prime}}}} \right) = \mathop \sum \limits_{k = 1}^{N} S(B_{i} |B_{k} ) = \mathop \sum \limits_{k = 1}^{N} ln\left( {\frac{{P\left( {B_{k} /B_{i} } \right)}}{{P\left( {B_{k} /\overline{{B_{i} }} } \right)}}} \right)$. In our case, $B_{i}$ is the set of cells with presence of each sand fly species, and $\overline{{B_{i} }}$ is the set of cells without presence of that sand fly species; $B_{k}$ are the number of cells of presence for each mammal order. The score function was implemented in MATLAB. The script loads the mammal and vector databases, and assigns each record to a cell using the collection coordinates. Then, it creates a 5 km buffer around each sand fly collection record and a 10 km buffer for each vertebrate record, and counts the number of cells where each sand fly species is present ($B_{i}$), not present ($\overline{{B_{i} }}$), and each mammal order is present ($B_{k}$) to find the most important statistical associations between vector and mammal distributions. Score values were computed for each sand fly species co-occurring with each mammal genus in each ecoregion (the code and databases are available at https://github.com/biomac-lab/leish).

To visualize the calculated co-occurrences, the sum of scores by the next taxonomic level (family) was calculated, and mammal families co-occurring with sand fly species were visualized in a heatmap for each ecoregion, using different scales of color according to the lowest and highest values. A hierarchical clustering analysis was performed based on Bray–Curtis distances, where clusters were calculated based on the co-occurrence similarities within sand flies and within mammals. The R software package ‘vegan’ was used to build the heatmaps with the dendrograms (Fig. S1).

**Figure 3:**
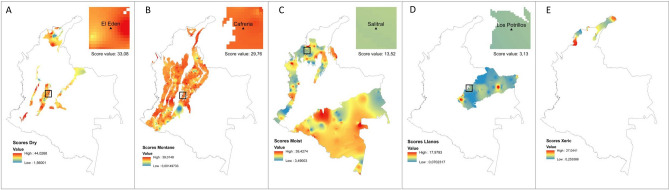
Predicted risk of sand fly presence based on the sum of score values by mammal genus, using the inverse distance weighted (IDW) method in ArcGis 10.7.1. (https://desktop.arcgis.com/en/arcmap/) Copyright 1995–2018 Esri. All rights reserved. Published in the United States of America.

In order to determine the spatial distribution of the scores as a proxy of the relative risk of sand fly presence per ecoregion, a score map based on the sum of mammal scores by genus was constructed^35^. The sum of all positive scores obtained for each genus in each ecoregion was assigned to the database of mammal occurrences and that was the data point used for interpolation. The interpolation was performed in ArcGis 10.7.1, using the inverse distance weighted (IDW) method, which determines cell values with a linearly weighted combination of a set of sample points; the weight is a function of inverse distance. This method assumes that the mapped scores decrease in influence with distance from the score value assigned to each occurrence. The parameters used were: power = 2, cell-size = 1 km^2^, and the extension and mask of the ecoregion. A positive score indicates there is a higher than random probability to find sand flies present^35^ (Fig. 3).

### Fieldwork co-occurrence consensus

To evaluate if sand fly species predicted by the score function to occur in each ecoregion were present, fieldwork was conducted in four sites, one per ecoregion, selected based on accessibility, and Leishmaniasis prevalence reports from the National System of Public Health Vigilance (SIVIGILA by its Spanish acronym)^36^ (Fig. 2). The selected sites were: Los Potrillos (Lowlands), Salitral (Moist Forest), Cafreria (Montane Forest), and El Eden (Dry Forest). The only ecoregion that could not be sampled was Xeric shrublands, primarily due to its low coverage in the country, and accessibility constrains (Fig. 3). Collection sites varied in the predicted score values obtained from the co-occurrence modeling (Fig. 3). For each field site, the sand fly species contributing to the score in that pixel (based on the IDW), were obtained and compared to the sand fly species collected. The comparison was performed using a correct classification index, using coincidences and divergences. Coincidences occurred when a sand fly species was collected in field and also predicted. Divergence occurred when either a sand fly species was collected but not predicted or it was predicted but not collected. Correct classification index was calculated as 2 × coincidences/2 × (coincidences + divergences), and it ranged from 0 to 1, being close to zero when there were very few coincidences or being close to one when there were several coincidences between observed and predicted species.

### Specimen collection and identification

Sand flies and mammals were collected in May, June and July 2016. Fieldwork captures were performed based on the web sampling designed by Parmenter et al.^37^, which includes 111 traps (87 Sherman, 24 Tomahawk) placed throughout 12 concentric transects. Each transect included 9 traps, the first three were placed 5 m apart from each other, and the distance between the following six was of 10 m. The 4th and 9th traps in each transect were Tomahawk, used for trapping medium-sized mammals, and the rest were Sherman traps used for small-sized mammals. Three more Sherman traps were placed in the center of the web. Sherman and Tomahawk traps were baited using a mixture of oatmeal, hazelnut chocolate cream, banana, wheat flour, granola, and canned tuna, and were set each morning after being checked for positive captures.

Insect miniature light traps from the Centers for Disease Control and Prevention (CDC) were placed between the concentric transects; each trap was hung on a tree 1 m above the ground, and was lit from 18:00 to 06:00 h. All traps were set for four consecutive nights.

Captured mammals were manipulated considering the adequate security measures in a field lab established for blood collection. In Los Potrillos and Salitral, individuals were anesthetized with intramuscular Zoletil (0.05 ml/40 g), and posteriorly weighted, measured (total length, tail length, feet length), sexed, and classified to juvenile or adult based on external genitalia^38^. Blood was collected by cardiac puncture using 0.5 ml sodium citrate in a 3-ml needle, and a sample of tissue was taken using an ear punch and stored in 70% ethanol. In Cafreria and El Eden, captured individuals were euthanized with Zoletil (0.5 ml/40 g), samples of liver, heart, spleen, and tissue were taken and stored in 90% ethanol. Euthanized individuals were processed to develop taxonomic identification and preserved in the Museum of Natural History of Universidad de Los Andes. Euthanized mammalian individuals were identified to species employing traditional taxonomic protocol, which is based on the identification of differential characters in the skull and jaws^38,39^.

Collected sand flies were kept in 70% ethanol and transported to the lab for taxonomic identification and molecular processing. Taxonomic identification was performed following Young and Duncan^40^ and Galati^41^. Species were identified considering the morphological features of the male and female genitalia, such as number of spines in the coxite in the case of males, and length and size of the spermatheca in females^2^. Male specimens were identified to species level using the previously mentioned protocol, but females were not dyed to preserve the DNA for molecular processing. In this case, the last part of the abdomen was cut to perform identification based only on the spermatheca, and the rest of the body was preserved in ethanol. From the total number of sand flies, 70% were selected to perform species identification.

### DNA extraction and amplification

Samples were processed in different ways depending on the analyses to be performed. For *Leishmania* detection, female, sand flies were pooled by species (up to 20 individuals per pool) to perform DNA extraction and amplification. To perform species identification through barcodes, sand flies and mammals were processed individually^2^. DNA extraction (50 ul) was performed using the High Pure PCR Template Preparation Kit from Roche following the manufacturer’s protocol.

### Leishmania detection

Samples from sand flies and mammals were processed using three sets of primers targeting HSP70 ITS and CytB genes^42–44^. The primers used were HSP70F (5′ AGG TGA AGG CGA CGA ACG 3′); HSP70R (5′ CGC TTG TCC ATC TTT GCG TC 3′) with an amplicon size of 337 bp and, LITSR (5′ CTG GAT CAT TTT CCG ATG 3′); L5.8S (5′ TGA TAC CAC TTA TCG CAC TT 3′) with an amplicon size of 300–350 bp, and primers CytBR (5′ AGC GGA GAG RAR AGA AAA GG 3′) and CytBF (5′ GYT CRC AAT AAA ATG CAA ATC 3′) with an amplicon size of 618 bp^45^. PCR amplification of these genes was performed using a master mix volume preparation of 25 ul containing 12.5 ul GoTaq *Green Master Mix* from Promega, 1.25 ul of each primer, 5 ul of nuclease free water and 5 ul of DNA. Thermal cycling conditions were performed as detailed by Hernández et al.^42^ for HSP70 and ITS; and for CytB^45^. *Leishmania* positive products were sequenced with HSP70 primers in both directions and Cytb primers with only the forward pair.

### Species identity confirmation

For species identity confirmation, DNA Barcoding was performed, using the primers LCO1490 (5′-GGT CAA CAA ATC ATA AAG ATA TTGG-3′) and HCO2198 (5′-TAA ACT TCA GGG TGA CCA AAA AAT CA-3′)^31,46–48^. PCR master mix was also prepared with the same volumes as previously mentioned and thermal cycling conditions were performed as detailed by Laurito et al*.*^47^.

Electrophoresis was run for all PCR products in a 2% agarose gel in 1× TBE Buffer at 125 V for 30 min. Gels were posteriorly visualized and recorded in the gel documentation system. Positive PCR products, or those in which a single band was visualized were sent to sequencing at the DNA Sequencing Center at Universidad de Los Andes.

Obtained sequences were cleaned and edited using Geneious 4.8.5^48^ and those with an HQ% lower than 50 were discarded. Consensus sequences were generated for each specimen and exported in Fasta for analysis. Each sequence was then compared with other nucleotide sequences by using the BLAST (Basic Local Alignment Search Tool) available in GenBank NCBI, to confirm the identity of the obtained fragments [https://blast.ncbi.nlm.nih.gov/Blast.cgi]. Sequences with a query coverage, and identity lower than 97% were discarded. DNA sequences alignment was performed in MEGA v7.0 software^45^ using the MUSCLE tool incorporated based on neighbor joining parameters. The inter and intraspecific sequence divergence were calculated using the distance model Kimura two-parameter (K2P), and a dendrogram of K2P distance journey was built in order to visualize sequence clustering patterns of the sequences obtained compared to reference sequences downloaded from the NCBI nucleotide Database^20^.

### Ethics statement

Sampling procedures were done under Universidad de Los Andes collection approval (Resolution 1177 October 9th 2014—IDB 0359) from the National Authority of Environmental Licenses (ANLA) and the ethical review board of the Universidad de los Andes (CICUAL–83 C.FUA 14-026).

## Supplementary Information

Supplementary Tables.

Supplementary Legends.

Supplementary Figure S1.

Supplementary Figure S2.

Supplementary Figure S3.

## References

[1] Bates PA (2015). Recent advances in phlebotomine sand fly research related to leishmaniasis control. Parasit. Vector.

[2] Ferro C (2011). Phlebotomine vector ecology in the domestic transmission of American cutaneous leishmaniasis in Chaparral, Colombia. Am. J. Trop. Med. Hyg..

[3] Maroli M, Feliciangeli MD, Bichaud L, Charrel RN, Gradoni LKM (2013). Phlebotomine sand flies and the spreading of leishmaniases and other diseases of public health concern. Med. Vet. Entomol..

[4] Velez ID, Hendrickx E, Robledo SM, Agudelo S (2001). Leishmaniosis cutánea en Colombia y género. Cad. Saude. Publ..

[5] WHO Department of Control of Neglected Tropical Diseases (2016). WHO: Weekly epidemiological record Relevé épidémiologique hebdomadaire. Wkly. Epidemiol. Rec..

[6] Ospina, S.A., Prieta, F.E., Pacheco, O. & Quijada, H. Boletin epidemiologico semanal. Preprint at https://www.ins.gov.co/buscador-eventos/BoletinEpidemiologico/2018%20Bolet%C3%ADn%20epidemiol%C3%B3gico%20semana%2031.pdf (2018)

[7] OMS (2010). Control de las leishmaniasis Informe de una reunión del comite de expertos de la OMS sobre el control de las Leishmaniasis. OMS.

[8] Agudelo, N.J. Informe de Evento Leishmaniasis Cutanea, Mucosa y Visceral, Colombia 2018. Preprint at https://www.ins.gov.co/buscador-eventos/Informesdeevento/LEISHMANIASIS_2018.pdf (2018)

[9] Ferro C (2015). Spatial distribution of sand fly vectors and eco-epidemiology of cutaneous leishmaniasis transmission in Colombia. PLoS ONE.

[10] Ramírez JD (2016). Taxonomy, diversity, temporal and geographical distribution of Cutaneous Leishmaniasis in Colombia: A retrospective study. Sci. Rep..

[11] Pardo RH, Cabrera OL, Becerra J, Fuya P, Ferro C (2006). *Lutzomyia longiflocosa*, posible vector en un foco de leishmaniasis cutánea en la región subandina del departamento del Tolima, Colombia. Biomedica.

[12] Ovalle C (2006). Distribución geográfica de especies de *Leishmania* aisladas de pacientes consultantes al Instituto Nacional de Dermatología Federico Lleras Acosta. Biomedica.

[13] Rodríguez-Barraquer I (2008). Etiologic agent of an epidemic of cutaneous leishmaniasis in Tolima, Colombia. Am. J. Trop. Med. Hyg..

[14] Alvar J (2012). Leishmaniasis worldwide and global estimates of its incidence. PLoS ONE.

[15] Bejarano EE, Estrada LG, Wolf M, Nihei S, Carvalho C (2016). Family Psychodidae. Catalogue of Diptera of Colombia.

[16] Roque ALR, Jansen AM (2014). Wild and synanthropic reservoirs of Leishmania species in the Americas. Int. J. Parasitol. Parasites Wildl..

[17] González C (2011). Current knowledge of Leishmania vectors in Mexico: How geographic distributions of species relate to transmission areas. Am. J. Trop. Med. Hyg..

[18] Stephens CR (2009). Using biotic interaction networks for prediction in biodiversity and emerging diseases. PLoS ONE.

[19] Urbano J (2011). Characterization of cutaneous isolates of *Leishmania* in Colombia by isoenzyme typing and kDNA restriction analysis. Rev. Ibero-latinoam..

[20] Contreras-Gutiérrez MA, Vélez ID, Porter C, Uribe SI (2014). Lista actualizada de flebotomíneos (Diptera: Psychodidae: Phlebotominae) de la región cafetera colombiana. Biomedica.

[21] Meyer CP, Paulay G (2005). DNA barcoding: Error rates based on comprehensive sampling. PLoS Biol..

[22] Desjeux P (1996). Leishmaniasis public health aspects and control. Clin. Dermatol..

[23] Ibarra-Cerdeña CN, Valiente-Banuet L, Sánchez-Cordero V, Stephens CR, Ramsey JM (2017). *Trypanosoma cruzi* reservoir—Triatomine vector co-occurrence networks reveal meta-community effects by synanthropic mammals on geographic dispersal. Peer J..

[24] Adler GH, Becerra MT, Travi BL (2003). Feeding success of *Lutzomyia evansi* (Diptera: Psychodidae) experimentally exposed to small mammal hosts in an endemic focus of Leishmania chagasi in northern Colombia. Biomedica.

[25] Adler GH, Arboledo JJ, Travi BL (1997). Diversity and abundance of small mammals in degraded tropical dry forest of northern Colombia. Mammalia.

[26] Travi BL (1994). *Didelphis marsupialis*, an important reservoir of *Trypanosoma (schizotrypanum) cruzi* and *Leishmania (leishmania) chagasi* in Colombia. Am. J. Trop. Med. Hyg..

[27] Pérez-Hernández, R., Ventura, J. & López Fuster, M. *Gracilinanus marica*. In *The IUCN Red List of Threatened Species*. 10.2305/IUCN.UK.20161.RLTS.T9420A22169944.en (2016).

[28] Berzunza-cruz M (2015). *Leishmania* (L.) *mexicana* infected bats in Mexico: Novel potential reservoirs. PLoS Negl. Trop. Dis..

[29] De Lima H (2008). Isolation and molecular identification of *Leishmania chagasi* from a bat (*Carollia perspicillata*) in northeastern Venezuela. Mem. Inst. Oswaldo. Cruz..

[30] Mouriz-Savani EM (2010). Veterinary parasitology detection of Leishmania (Leishmania) amazonensis and Leishmania (Leishmania) infantum chagasi in Brazilian bats. Vet. Parasitol..

[31] Sousa LCC, Gontijo CMF, Botelho HA, Fonseca CG (2012). Mitochondrial genetic variability of *Didelphis albiventris* (Didelphimorphia, Didelphidae) in Brazilian localities. Genet. Mol. Biol..

[32] De Rezende MB (2017). Detection of Leishmania spp in bats from an area of Brazil endemic for visceral leishmaniasis. Transbound Emerg Dis..

[33] Killick-Kendrick R (1990). Phlebotomine vectors of the leishmaniases: A review. Med. Vet. Entomol..

[34] Ponce N, Zipa Y, Ferro C (2006). Presencia en el peridomicilio de vectores infectados con *Leishmania* (Viannia) *panamensis* en dos focos endémicos en el occidente de Boyacá, piedemonte del valle del Magdalena medio, Colombia. Biomedica.

[35] Stephens CR (2016). Can you judge a disease host by the company it keeps? Predicting disease hosts and their relative importance: A case study for leishmaniasis. PLoS. Negl. Trop. Dis..

[36] Sistema Nacional de Vigilancia en Salud Pública—SIVIGILA. Reportes epidemiológicos (2016). http://www.ins.gov.co/lineas-deaccion/SubdireccionVigilancia/sivigila/Paginas/sivigila.aspx. Accessed July 2019.

[37] Parmenter RR (2002). Small-mammal density estimation: a field comparison of grid-based vs. web-based density estimators. Ecol. Monogr..

[38] Tirira D (1998). Tecnicas de Campo Para el Estudio de Mamiferos Silvestres. Potinficia Universidad Catolica del Ecuador/SIMBIOE. Publ. Especial Quito.

[39] Cuartas-Calle C, Muñoz-Arango J (2003). Marsupiales, Celonéstidos, e insectivoros de Colombia. Edit. Univ. de Antioquia (Medellín).

[40] Young D, Duncan M (1994). Guide to the identification and geographic distribution of *Lutzomyia* sand flies in Mexico, the West Indies, Central and South America (Diptera: Psychodidae).

[41] Galati EAB (2015). Apostila de Bioecologia e Identificação de Phlebotominae (Diptera, Psychodidae).

[42] Ocampo CB (2012). Environmental factors associated with American cutaneous leishmaniasis in a new Andean focus in Colombia. Trop. Med. Int. Health.

[43] Hernández C (2014). Identification of Six New World *Leishmania* species through the implementation of a high-resolution melting (HRM) genotyping assay. Parasit. Vectors.

[44] El Tail NO, Osman OF, El Fari M, Presber W, Schonian G (1996). Genetic heterogeneity of ribosomal internal transcribed spacer in clinical samples of *Leishmania donovani* spotted on filter paper as revealed by single-strand conformation polymorphisms and sequencing. Trans. R. Soc. Trop. Med. Hyg..

[45] Herrera G (2017). Evaluation of a multilocus sequence typing (MLST) scheme for *Leishmania* (Viannia) *braziliensis* and *Leishmania* (Viannia) *panamensis* in Colombia. Parasit. Vectors.

[46] Molaei G, Andreadis TG, Armstrong PM, Anderson JF, Vossbrinck CR (2006). Host feeding patterns of *Culex* mosquitoes and west nile virus transmission, northeastern United States. Emerg. Infect. Dis..

[47] Laurito M, De Oliveira TMP, Almirón WR, Sallum MAM (2013). COI barcode versus morphological identification of *Culex* (Culex) (Diptera: Culicidae) species: A case study using samples from Argentina and Brazil. Mem. Inst. Oswaldo. Cruz..

[48] Kearse M (2012). Geneious Basic: An integrated and extendable desktop software platform for the organization and analysis of sequence data. Bioinformatics.

