# Supplementary material for: Measuring spatial co-occurrences of species potentially involved in Leishmania transmission cycles through a predictive and fieldwork approach
Source: Sci Rep. 2021 Mar 24;11:6789. doi: 10.1038/s41598-021-85763-9 (PMC7990927; doi:10.1038/s41598-021-85763-9)
Supplement: Supplementary file 1 — Supplementary Tables. [file 41598_2021_85763_MOESM1_ESM.docx]

**Measuring spatial co-occurrences to infer species potentially involved in *Leishmania* transmission cycles through a modeling and fieldwork approach**

Marla López^1^, Diana Erazo^2^, Juliana Hoyos^1^, Cielo León^1^, Patricia Fuya^3^, Ligia Lugo^3^, Juan Manuel Cordovez^2^, Camila González^1*^

^1^Centro de Investigaciones en Microbiología y Parasitología Tropical (CIPAT), Facultad de Ciencias, Departamento de Ciencias Biológicas, Universidad de los Andes, Bogotá – Colombia.

^2^Grupo de investigación en Biología Matemática y Computacional, Facultad de Ingeniería, Departamento de Ingeniería Civil y Ambiental, Universidad de los Andes, Bogotá - Colombia.

^3^Grupo de Entomología, Instituto Nacional de Salud (INS), Bogotá, Colombia.

*Correspondence author: Camila González, Centro de Investigaciones en Microbiología y Parasitología Tropical (CIMPAT), Facultad de Ciencias, Departamento de Ciencias Biológicas, Universidad de los Andes, Bogotá – Colombia. E-mail: c.gonzalez2592@uniandes.edu.co

Table S1. Mammalian species identified by sequencing of PCR templates obtained from amplification with COI primers.

| Collection site | Description (species) | Accesion code | Identity (%) | E-value |
| --- | --- | --- | --- | --- |
| Los Potrillos | *Zygodontomys brevicauda* | [JF492746.1](https://www.ncbi.nlm.nih.gov/nucleotide/328483536?report=genbank&log$=nucltop&blast_rank=1&RID=A6UY16A9015) | 99 | 0 |
| Los Potrillos | *Zygodontomys brevicauda* | [JF492746.1](https://www.ncbi.nlm.nih.gov/nucleotide/328483536?report=genbank&log$=nucltop&blast_rank=1&RID=A6VDC9F1015) | 99 | 0 |
| Los Potrillos | *Zygodontomys brevicauda* | [JF492746.1](https://www.ncbi.nlm.nih.gov/nucleotide/328483536?report=genbank&log$=nucltop&blast_rank=1&RID=A6VHRRB5015) | 99 | 0 |
| Los Potrillos | *Zygodontomys brevicauda* | [JF492746.1](https://www.ncbi.nlm.nih.gov/nucleotide/328483536?report=genbank&log$=nucltop&blast_rank=1&RID=A6VM47YY014) | 99 | 0 |
| Los Potrillos | *Zygodontomys brevicauda* | [JF492746.1](https://www.ncbi.nlm.nih.gov/nucleotide/328483536?report=genbank&log$=nucltop&blast_rank=1&RID=A6W1HFP1014) | 99 | 0 |
| Los Potrillos | *Zygodontomys brevicauda* | [JF492746.1](https://www.ncbi.nlm.nih.gov/nucleotide/328483536?report=genbank&log$=nucltop&blast_rank=1&RID=A6W8NK8Z014) | 99 | 0 |
| Los Potrillos | *Plathyrrinus brachicephalus* | [JF447853.1](https://www.ncbi.nlm.nih.gov/nucleotide/326700510?report=genbank&log$=nucltop&blast_rank=1&RID=AC21524M014) | 99 | 0 |
| Los Potrillos | *Zygodontomys brevicauda* | [JF492746.1](https://www.ncbi.nlm.nih.gov/nucleotide/328483536?report=genbank&log$=nucltop&blast_rank=1&RID=AC27ZGK4015) | 99 | 0 |
| Los Potrillos | *Myotis riparius* | [JQ601617.1](https://www.ncbi.nlm.nih.gov/nucleotide/379744650?report=genbank&log$=nucltop&blast_rank=2&RID=AC29EXTT014) | 99 | 0 |
| Los Potrillos | *Myotis riparius* | [JQ601627.1](https://www.ncbi.nlm.nih.gov/nucleotide/379744670?report=genbank&log$=nucltop&blast_rank=1&RID=AC2K9HBH015) | 99 | 0 |
| Los Potrillos | *Zygodontomys brevicauda* | [JF492746.1](https://www.ncbi.nlm.nih.gov/nucleotide/328483536?report=genbank&log$=nucltop&blast_rank=1&RID=AC27ZGK4015) | 96 | 0 |
| Los Potrillos | *Carollia perspicillata* | [HG003309.1](https://www.ncbi.nlm.nih.gov/nucleotide/543172561?report=genbank&log$=nucltop&blast_rank=1&RID=AC2UBX9K014) | 99 | 0 |
| Los Potrillos | *Carollia perspicillata* | [HG003309.1](https://www.ncbi.nlm.nih.gov/nucleotide/543172561?report=genbank&log$=nucltop&blast_rank=1&RID=AC2UBX9K014) | 99 | 0 |
| Los Potrillos | *Lophostoma brasiliense* | [EF080424.1](https://www.ncbi.nlm.nih.gov/nucleotide/117936926?report=genbank&log$=nucltop&blast_rank=1&RID=AC3314VA014) | 98 | 0 |
| Los Potrillos | *Zygodontomys brevicauda* | [JF492747.1](https://www.ncbi.nlm.nih.gov/nucleotide/328483538?report=genbank&log$=nucltop&blast_rank=1&RID=AVGFA3U3014) | 98 | 0 |
| Los Potrillos | *Zygodontomys brevicauda* | [JF492746.1](https://www.ncbi.nlm.nih.gov/nucleotide/328483536?report=genbank&log$=nucltop&blast_rank=1&RID=AC27ZGK4015) | 96 | 0 |
| Los Potrillos | *Zygodontomys brevicauda* | [JF492746.1](https://www.ncbi.nlm.nih.gov/nucleotide/328483536?report=genbank&log$=nucltop&blast_rank=1&RID=AC27ZGK4015) | 98 | 0 |
| Los Potrillos | *Zygodontomys brevicauda* | [JF492746.1](https://www.ncbi.nlm.nih.gov/nucleotide/328483536?report=genbank&log$=nucltop&blast_rank=1&RID=AC27ZGK4015) | 99 | 0 |
| Los Potrillos | *Carollia perspicillata* | [JF447802.1](https://www.ncbi.nlm.nih.gov/nucleotide/326700408?report=genbank&log$=nucltop&blast_rank=1&RID=AVGW9SP301R) | 99 | 0 |
| Los Potrillos | Zygodontomys brevicauda | [JF492746.1](https://www.ncbi.nlm.nih.gov/nucleotide/328483536?report=genbank&log$=nucltop&blast_rank=1&RID=AC27ZGK4015) | 99 | 0 |
| Los Potrillos | *Carollia perspicillata* | JF447609.1 | 99 | 0 |
| Los Potrillos | *Zygodontomys brevicauda* | [JF492746.1](https://www.ncbi.nlm.nih.gov/nucleotide/328483536?report=genbank&log$=nucltop&blast_rank=1&RID=AC27ZGK4015) | 99 | 0 |
| Los Potrillos | *Zygodontomys brevicauda* | [JF492746.1](https://www.ncbi.nlm.nih.gov/nucleotide/328483536?report=genbank&log$=nucltop&blast_rank=1&RID=AC27ZGK4015) | 99 | 0 |
| Los Potrillos | *Carollia perspicillata* | [EU096652.1](https://www.ncbi.nlm.nih.gov/nucleotide/156078721?report=genbank&log$=nucltop&blast_rank=2&RID=AVHNGF6V015) | 98 | 0 |
| Los Potrillos | *Zygodontomys brevicauda* | [JF492746.1](https://www.ncbi.nlm.nih.gov/nucleotide/328483536?report=genbank&log$=nucltop&blast_rank=1&RID=AC27ZGK4015) | 99 | 0 |
| Los Potrillos | *Zygodontomys brevicauda* | [JF492746.1](https://www.ncbi.nlm.nih.gov/nucleotide/328483536?report=genbank&log$=nucltop&blast_rank=1&RID=AC27ZGK4015) | 99 | 0 |
| Los Potrillos | *Artibeus planirostris* | [JF448664.1](https://www.ncbi.nlm.nih.gov/nucleotide/327203129?report=genbank&log$=nucltop&blast_rank=1&RID=AVP64R37014) | 99 | 0 |
| Los Potrillos | *Zygodontomys brevicauda* | [JF492746.1](https://www.ncbi.nlm.nih.gov/nucleotide/328483536?report=genbank&log$=nucltop&blast_rank=1&RID=AC27ZGK4015) | 99 | 0 |
| Los Potrillos | *Phyllostomus discolor* | [EF080546.1](https://www.ncbi.nlm.nih.gov/nucleotide/117937170?report=genbank&log$=nucltop&blast_rank=1&RID=AVPH8KKS015) | 99 | 0 |
| Los Potrillos | *Plathyrrinus brachicephalus* | [JF447853.1](https://www.ncbi.nlm.nih.gov/nucleotide/326700510?report=genbank&log$=nucltop&blast_rank=1&RID=AVPHUYR7014) | 99 | 0 |
| Los Potrillos | *Plathyrrinus brachicephalus* | [JF447853.1](https://www.ncbi.nlm.nih.gov/nucleotide/326700510?report=genbank&log$=nucltop&blast_rank=1&RID=AVPHUYR7014) | 99 | 0 |
| Los Potrillos | *Zygodontomys brevicauda* | [JF492746.1](https://www.ncbi.nlm.nih.gov/nucleotide/328483536?report=genbank&log$=nucltop&blast_rank=1&RID=AC27ZGK4015) | 99 | 0 |
| Los Potrillos | *Zygodontomys brevicauda* | [JF492746.1](https://www.ncbi.nlm.nih.gov/nucleotide/328483536?report=genbank&log$=nucltop&blast_rank=1&RID=AC27ZGK4015) | 99 | 0 |

Table S2. Phlebotomine sand fly species identified by sequencing of PCR templates obtained from amplification with COI primers. Species names are written as found in Blast.

| **Sequence Code** | **Collection site** | **Description (species)** | **Accesion code** | **Identity (%)** | **E-value** |
| --- | --- | --- | --- | --- | --- |
| I2A1 | Los Potrillos | *Lutzomyia trinidadensis* | [JN845559.1](https://www.ncbi.nlm.nih.gov/nucleotide/402715598?report=genbank&log$=nucltop&blast_rank=5&RID=A6WTEG7X015) | 99 | 0 |
| 242 | Los Potrillos | *Psychodopygus panamensis* | [KC921266.1](https://www.ncbi.nlm.nih.gov/nucleotide/546605256?report=genbank&log$=nucltop&blast_rank=1&RID=A6Y47UYY014) | 89 | 0 |
| C3L | El Eden | *Lutzoymia longipalpis* | [GU909505.1](https://www.ncbi.nlm.nih.gov/nucleotide/304435355?report=genbank&log$=nucltop&blast_rank=1&RID=A8NFY3AU015) | 98 | 0 |
| C33L | Cafreria | *Lutzomyia pia* | [KC921267.1](https://www.ncbi.nlm.nih.gov/nucleotide/546605258?report=genbank&log$=nucltop&blast_rank=1&RID=A8P4ZAYH015) | 93 | 0 |
| C42L | Cafreria | *Lutzomyia pia* | [KC921267.1](https://www.ncbi.nlm.nih.gov/nucleotide/546605258?report=genbank&log$=nucltop&blast_rank=1&RID=A8P4ZAYH015) | 93 | 0 |
| C52L | Cafreria | *Lutzomyia pia* | [KC921267.1](https://www.ncbi.nlm.nih.gov/nucleotide/546605258?report=genbank&log$=nucltop&blast_rank=1&RID=A8R68MYE015) | 93 | 0 |
| C53L | Cafreria | *Lutzomyia trinidadensis* | [JN845559.1](https://www.ncbi.nlm.nih.gov/nucleotide/402715598?report=genbank&log$=nucltop&blast_rank=5&RID=A8RD0J6B015) | 98 | 0 |
| C73L | Cafreria | *Lutzomyia trinidadensis* | [JN845559.1](https://www.ncbi.nlm.nih.gov/nucleotide/402715598?report=genbank&log$=nucltop&blast_rank=5&RID=A91XZ00T01R) | 99 | 0 |
| G4 | Los Potrillos | *Lutzomyia trinidadensis* | [JN845559.1](https://www.ncbi.nlm.nih.gov/nucleotide/402715598?report=genbank&log$=nucltop&blast_rank=5&RID=A91XZ00T01R) | 99 | 0 |
| H2NCO | El Eden | *Lutzomyia trinidadensis* | [JN845559.1](https://www.ncbi.nlm.nih.gov/nucleotide/402715598?report=genbank&log$=nucltop&blast_rank=5&RID=A91XZ00T01R) | 97 | 0 |
| H3L | El Eden | *Lutzomyia trinidadensis* | [JN845559.1](https://www.ncbi.nlm.nih.gov/nucleotide/402715598?report=genbank&log$=nucltop&blast_rank=5&RID=A91XZ00T01R) | 96 | 0 |
| IC10 | Cafreria | *Lutzomyia longipalpis* | [JN845541.1](https://www.ncbi.nlm.nih.gov/nucleotide/402715562?report=genbank&log$=nucltop&blast_rank=1&RID=A935HKSJ01R) | 99 | 0 |
| IC51 | Cafreria | *Psychodopygus panamensis* | [GU909460.1](https://www.ncbi.nlm.nih.gov/nucleotide/304435265?report=genbank&log$=nucltop&blast_rank=1&RID=A936DYC301R) | 95 | 0 |
| IC53 | Cafreria | *Lutzomyia longipalpis* | [JN845542.1](https://www.ncbi.nlm.nih.gov/nucleotide/402715564?report=genbank&log$=nucltop&blast_rank=1&RID=A93N1HCK015) | 99 | 0 |
| IC62 | Cafreria | *Lutzomyia trinidadensis* | [JN845557.1](https://www.ncbi.nlm.nih.gov/nucleotide/402715594?report=genbank&log$=nucltop&blast_rank=5&RID=A98TWMRM015) | 98 | 0 |
| IC72 | Cafreria | *Lutzomyia longipalpis* | [GU909505.1](https://www.ncbi.nlm.nih.gov/nucleotide/304435355?report=genbank&log$=nucltop&blast_rank=1&RID=A9965SMM014) | 97 | 0 |
| IC73 | Cafreria | *Lutzomyia torvida* | [FJ437275.1](https://www.ncbi.nlm.nih.gov/nucleotide/219663913?report=genbank&log$=nucltop&blast_rank=1&RID=A99EW1D8014) | 99 | 0 |
| IC82 | Cafreria | *Lutzomyia longipalpis* | [GU909505.1](https://www.ncbi.nlm.nih.gov/nucleotide/304435355?report=genbank&log$=nucltop&blast_rank=1&RID=A9965SMM014) | 97 | 0 |
| NA1 | El Eden | *Lutzomyia trinidadensis* | [JN845559.1](https://www.ncbi.nlm.nih.gov/nucleotide/402715598?report=genbank&log$=nucltop&blast_rank=5&RID=A9AFW4W9014) | 99 | 0 |
| NA1PCO | El Eden | *Lutzomyia trinidadensis* | [JN845555.1](https://www.ncbi.nlm.nih.gov/nucleotide/402715590?report=genbank&log$=nucltop&blast_rank=5&RID=A9AGH3Y8015) | 96 | 0 |
| NA3 | El Eden | *Lutzomyia trinidadensis* | [KC921306.1](https://www.ncbi.nlm.nih.gov/nucleotide/546605336?report=genbank&log$=nucltop&blast_rank=1&RID=A9BC228A014) | 96 | 0 |
| ND1 | El Eden | *Lutzomyia trinidadensis* | [KC921306.1](https://www.ncbi.nlm.nih.gov/nucleotide/546605336?report=genbank&log$=nucltop&blast_rank=1&RID=A9BCNB3S015) | 96 | 0 |
| ND1L | El Eden | *Lutzomyia trinidadensis* | [JN845555.1](https://www.ncbi.nlm.nih.gov/nucleotide/402715590?report=genbank&log$=nucltop&blast_rank=5&RID=A9AGH3Y8015) | 96 | 0 |
| ND1PCO | El Eden | *Lutzomyia trinidadensis* | [JN845555.1](https://www.ncbi.nlm.nih.gov/nucleotide/402715590?report=genbank&log$=nucltop&blast_rank=5&RID=A9AGH3Y8015) | 97 | 0 |
| ND2 | El Eden | *Lutzomyia trinidadensis* | [JN845555.1](https://www.ncbi.nlm.nih.gov/nucleotide/402715590?report=genbank&log$=nucltop&blast_rank=5&RID=A9AGH3Y8015) | 96 | 0 |
| ND3 | El Eden | *Lutzomyia longipalpis* | [JN845542.1](https://www.ncbi.nlm.nih.gov/nucleotide/402715564?report=genbank&log$=nucltop&blast_rank=1&RID=A9CED12C014) | 99 | 0 |
| ND4 | El Eden | *Lutzomyia longipalpis* | [JN845542.1](https://www.ncbi.nlm.nih.gov/nucleotide/402715564?report=genbank&log$=nucltop&blast_rank=1&RID=A9CED12C014) | 99 | 0 |
| ND5 | El Eden | *Lutzomyia trinidadensis* | [JN845557.1](https://www.ncbi.nlm.nih.gov/nucleotide/402715594?report=genbank&log$=nucltop&blast_rank=2&RID=A9DDAJV5014) | 89 | 1,00E-180 |
| NE1 | El Eden | *Micropigomyia cayennensis* | [KR907863.1](https://www.ncbi.nlm.nih.gov/nucleotide/983176121?report=genbank&log$=nucltop&blast_rank=4&RID=A9DDWEJ6015) | 99 | 0 |
| NE1L | El Eden | *Lutzomyia longipalpis* | [JN845542.1](https://www.ncbi.nlm.nih.gov/nucleotide/402715564?report=genbank&log$=nucltop&blast_rank=1&RID=A9DYHFHG01R) | 99 | 0 |
| NE2 | El Eden | *Lutzomyia longipalpis* | [JN845542.1](https://www.ncbi.nlm.nih.gov/nucleotide/402715564?report=genbank&log$=nucltop&blast_rank=1&RID=A9DYHFHG01R) | 99 | 0 |
| NE2L | El Eden | *Lutzomyia trinidadensis* | [KC921306.1](https://www.ncbi.nlm.nih.gov/nucleotide/546605336?report=genbank&log$=nucltop&blast_rank=1&RID=A9E90WCE01R) | 96 | 0 |
| NE4 | El Eden | *Lutzomyia trinidadensis* | [JN845557.1](https://www.ncbi.nlm.nih.gov/nucleotide/402715594?report=genbank&log$=nucltop&blast_rank=5&RID=A9EA19RF01R) | 96 | 0 |
| NE5 | El Eden | *Lutzomyia longipalpis* | [JN845542.1](https://www.ncbi.nlm.nih.gov/nucleotide/402715564?report=genbank&log$=nucltop&blast_rank=1&RID=A9CED12C014) | 99 | 0 |
| NE6 | El Eden | *Lutzomyia longipalpis* | [JN845542.1](https://www.ncbi.nlm.nih.gov/nucleotide/402715564?report=genbank&log$=nucltop&blast_rank=1&RID=A9CED12C014) | 99 | 0 |
| NF1 | El Eden | *Lutzomyia longipalpis* | GU909505.1 | 98 | 0 |
| NF3 | El Eden | *Lutzomyia cayennensis cayennensis* | [GU909475.1](https://www.ncbi.nlm.nih.gov/nucleotide/304435295?report=genbank&log$=nucltop&blast_rank=1&RID=A9F9ZT6D01R) | 98 | 0 |
| NF6 | El Eden | *Lutzomyia longipalpis* | [JN845542.1](https://www.ncbi.nlm.nih.gov/nucleotide/402715564?report=genbank&log$=nucltop&blast_rank=1&RID=A9CED12C014) | 99 | 0 |
| NF7 | El Eden | *Lutzomyia trinidadensis* | [JN845557.1](https://www.ncbi.nlm.nih.gov/nucleotide/402715594?report=genbank&log$=nucltop&blast_rank=5&RID=A9EA19RF01R) | 98 | 0 |
| NI2 | El Eden | *Lutzomyia trinidadensis* | [KC921306.1](https://www.ncbi.nlm.nih.gov/nucleotide/546605336?report=genbank&log$=nucltop&blast_rank=1&RID=A9E90WCE01R) | 96 | 0 |
| NI3 | El Eden | *Lutzomyia longipalpis* | GU909505.1 | 98 | 0 |
| NI4 | El Eden | *Lutzomyia longipalpis* | GU909505.1 | 97 | 0 |
| NI5 | El Eden | *Lutzomyia longipalpis* | [JN845542.1](https://www.ncbi.nlm.nih.gov/nucleotide/402715564?report=genbank&log$=nucltop&blast_rank=1&RID=A9CED12C014) | 99 | 0 |
| NI6 | El Eden | *Lutzomyia longipalpis* | GU909505.1 | 98 | 0 |
| NJ1L | El Eden | *Lutzoymia trinidadensis* | [KC921306.1](https://www.ncbi.nlm.nih.gov/nucleotide/546605336?report=genbank&log$=nucltop&blast_rank=1&RID=ABETMN1X01R) | 96 | 0 |
| NJ2 | El Eden | *Lutzomyia longipalpis* | GU909505.1 | 98 | 0 |
| NJ5 | El Eden | *Lutzomyia longipalpis* | [JN845542.1](https://www.ncbi.nlm.nih.gov/nucleotide/402715564?report=genbank&log$=nucltop&blast_rank=1&RID=A9CED12C014) | 99 | 0 |
| XN2CO | El Eden | *Micropigomyia cayennensis* | [GU909473.1](https://www.ncbi.nlm.nih.gov/nucleotide/304435291?report=genbank&log$=nucltop&blast_rank=1&RID=ABMJEGYG015) | 99 | 0 |
| XN3CO | El Eden | *Lutzomyia trinidadensis* | [GU909498.1](https://www.ncbi.nlm.nih.gov/nucleotide/304435341?report=genbank&log$=nucltop&blast_rank=1&RID=ABMK3EN4015) | 96 | 0 |
| XN6CO | El Eden | *Lutzomyia longipalpis* | GU909505.1 | 98 | 0 |
| XN7CO | El Eden | *Lutzomyia trinidadensis* | [KC921306.1](https://www.ncbi.nlm.nih.gov/nucleotide/546605336?report=genbank&log$=nucltop&blast_rank=1&RID=ABS7KRXB01R) | 94 | 0 |
