# Supplementary material for: Measuring spatial co-occurrences of species potentially involved in Leishmania transmission cycles through a predictive and fieldwork approach
Source: Sci Rep. 2021 Mar 24;11:6789. doi: 10.1038/s41598-021-85763-9 (PMC7990927; doi:10.1038/s41598-021-85763-9)
Supplement: Supplementary file 2 — Supplementary Legends. [file 41598_2021_85763_MOESM2_ESM.docx]

**Figure S1.** (Panels A – E). Heat maps and hierarchical clustering (Bray-Curtis) of each ecoregion based on the scores calculated for each co-occurrence event. The color key for the heatmap is shown in the left superior corner, indicating the range of score values. The heatmaps were performed for each ecoregion, and distribution records for sand flies and mammals are shown (A) Dry forest, (B) Moist forest, (C) Montane forest, (D) Lowlands, (E) Xeric shrublands. Created in ArcGis 10.7.1. (https://desktop.arcgis.com/en/arcmap/) Copyright 1995-2018 Esri. All rights reserved. Published in the United States of America

**Figure S2.** Genetic distances. Frequency histograms of K2P genetic divergence distances calculated between Phlebotominae sand fly species COI sequences from collections in all studies sites.

**Figure S3.** K2P neighbor joining dendrogram of the sand fly species sequences obtained compared with reference sequences from GenBank. Reference sequences are grouped and identified with a triangle, n= indicates the number of sequences used per sand fly species.
