# Supplementary figures and images for: Measuring spatial co-occurrences of species potentially involved in Leishmania transmission cycles through a predictive and fieldwork approach
Source: Sci Rep. 2021 Mar 24;11:6789. doi: 10.1038/s41598-021-85763-9 (PMC7990927; doi:10.1038/s41598-021-85763-9)

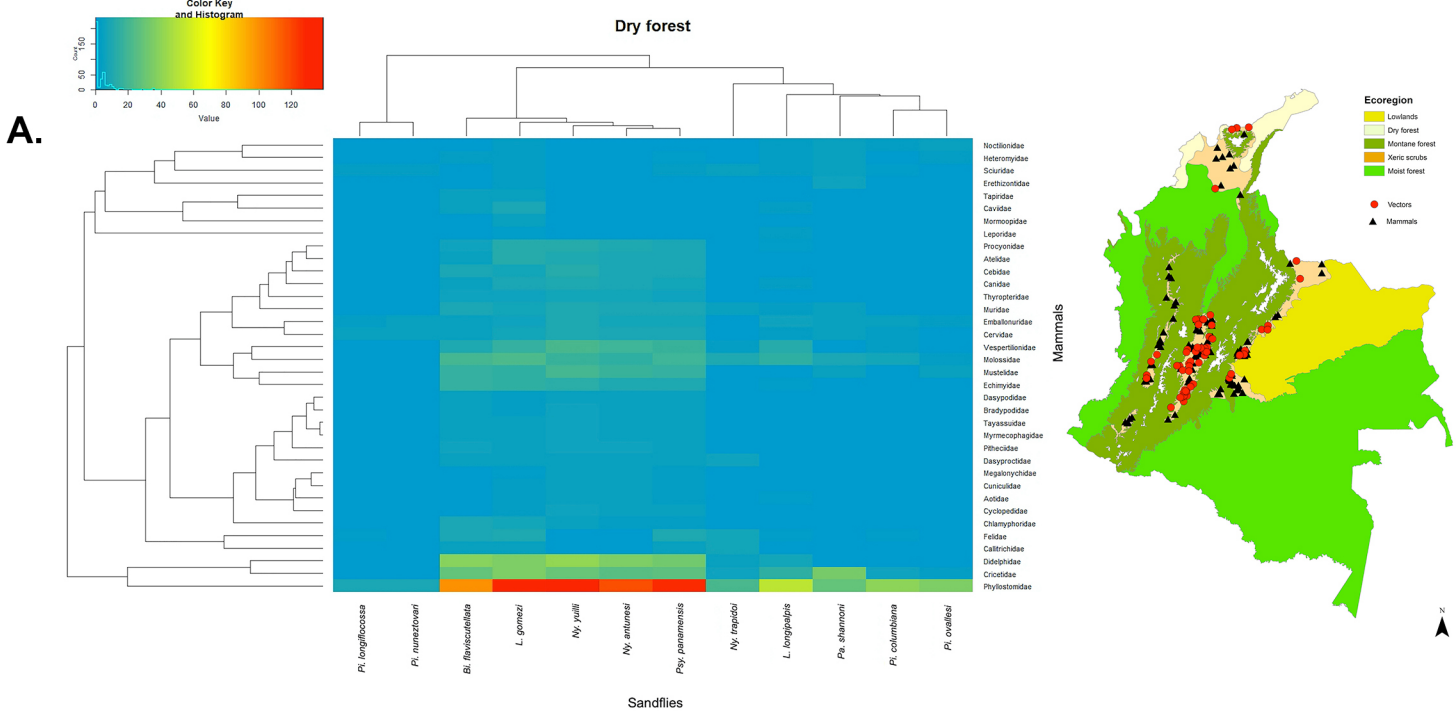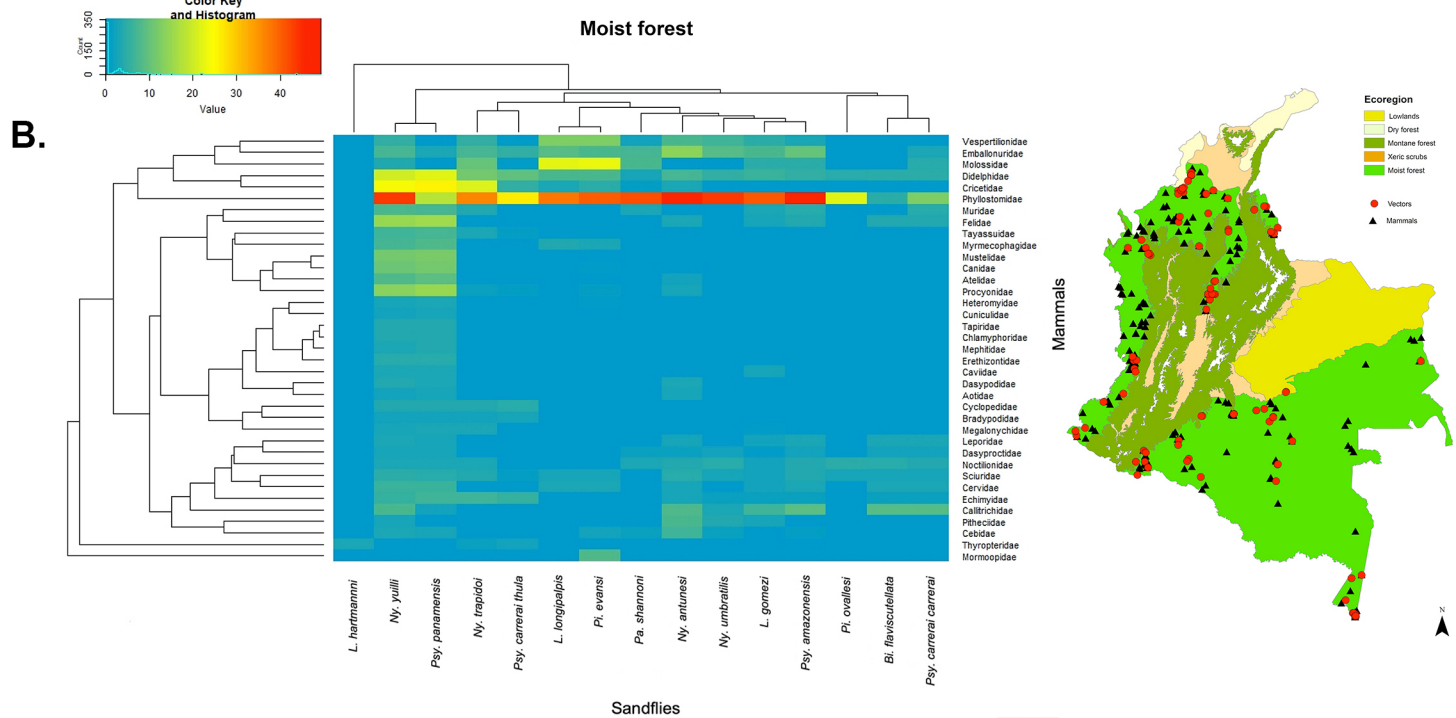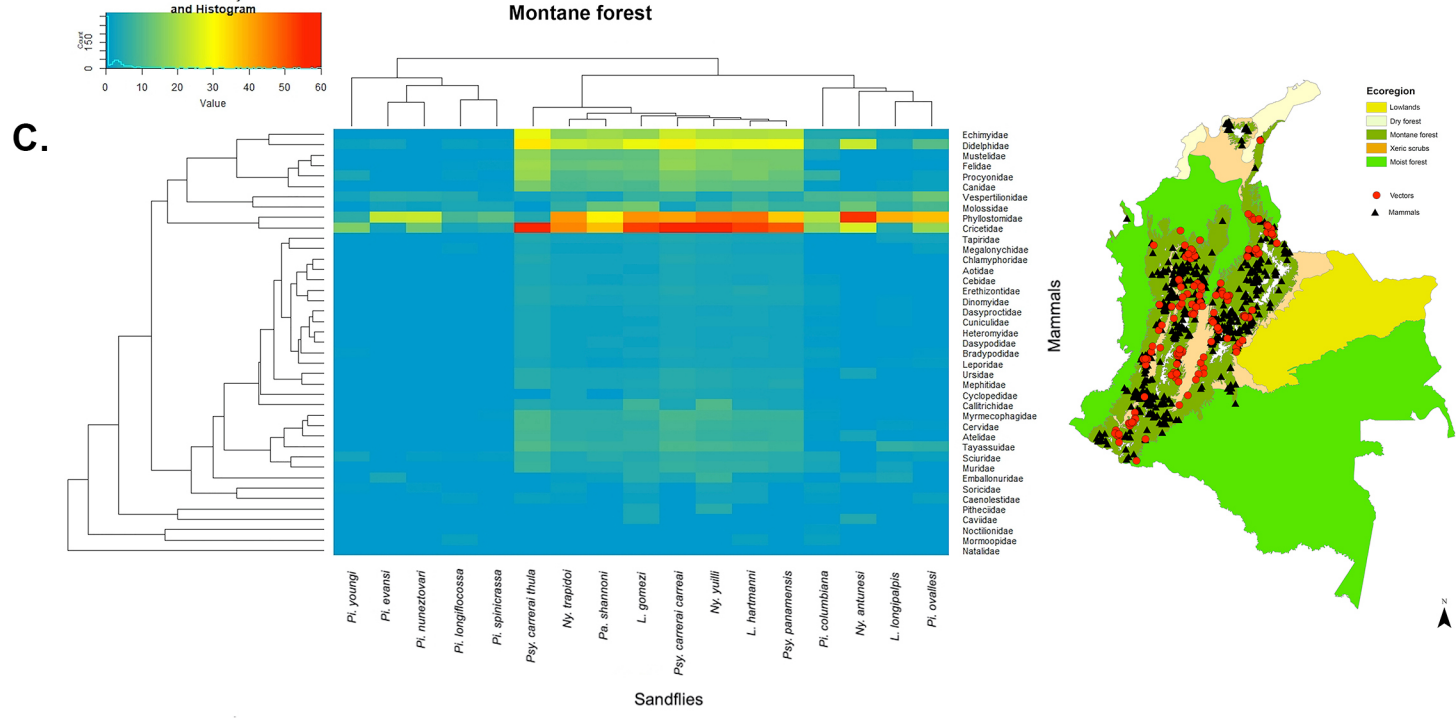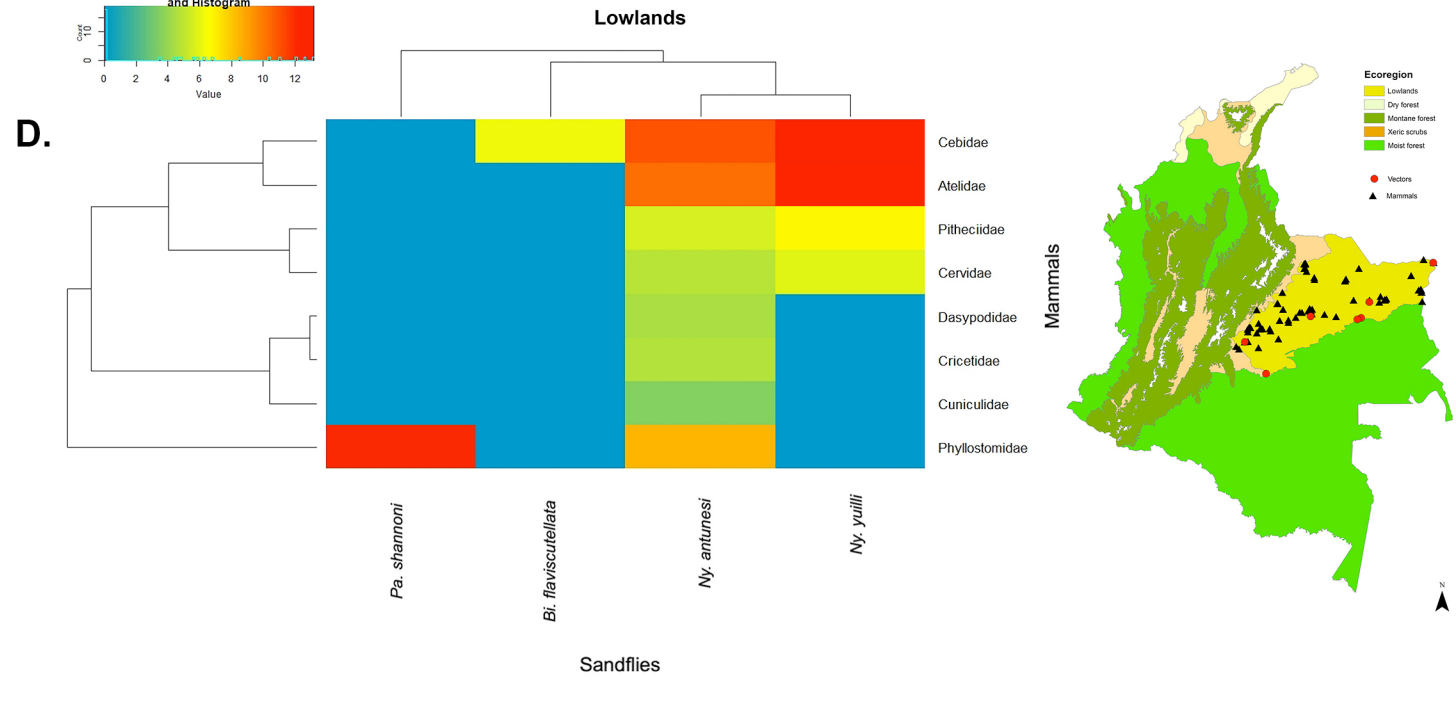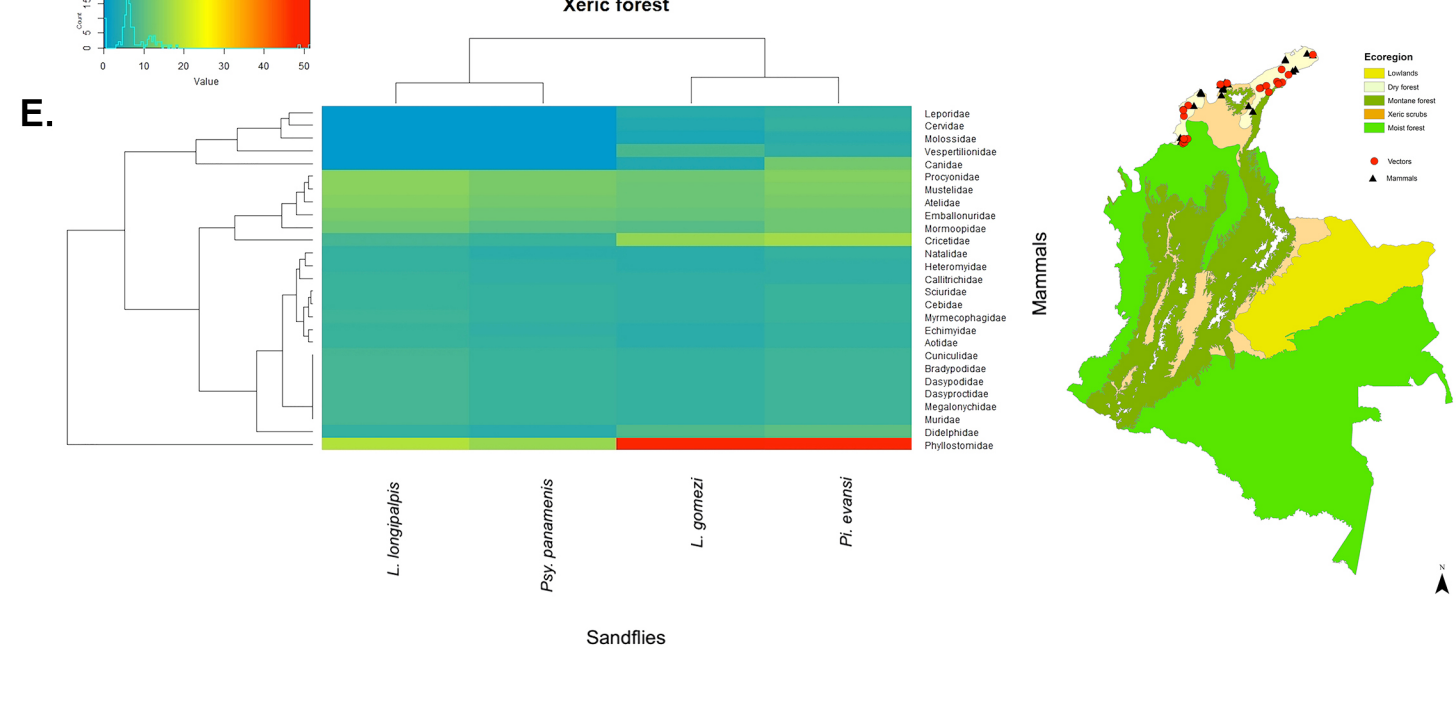

Supplement: Supplementary file 3 — Supplementary Figure S1. [file 41598_2021_85763_MOESM3_ESM.pdf]

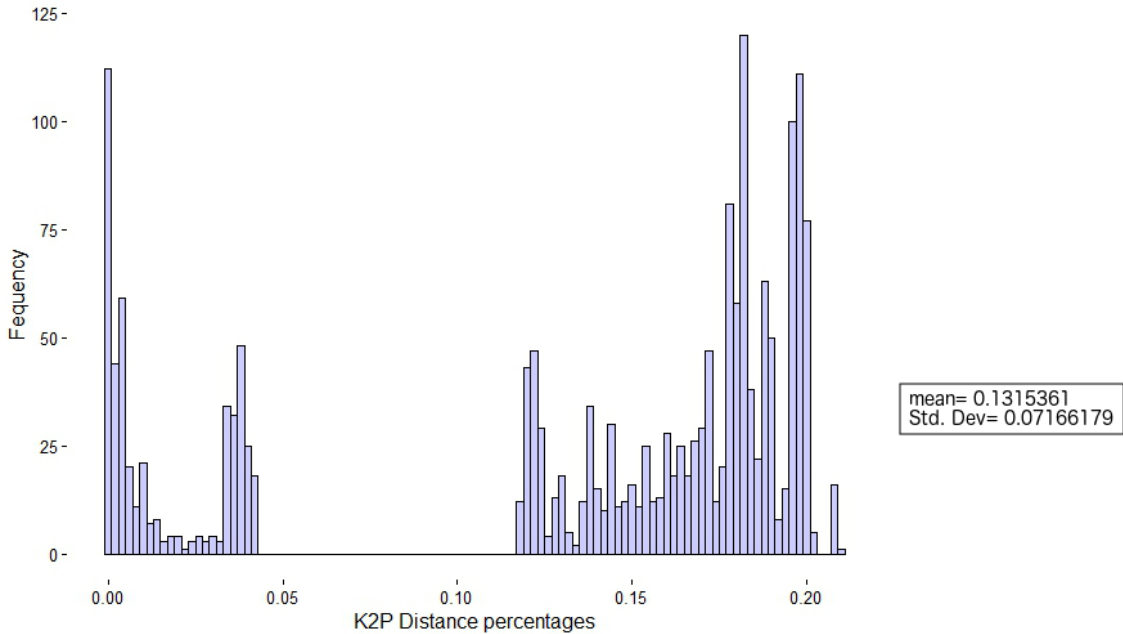

Supplement: Supplementary file 4 — Supplementary Figure S2. [file 41598_2021_85763_MOESM4_ESM.pdf]

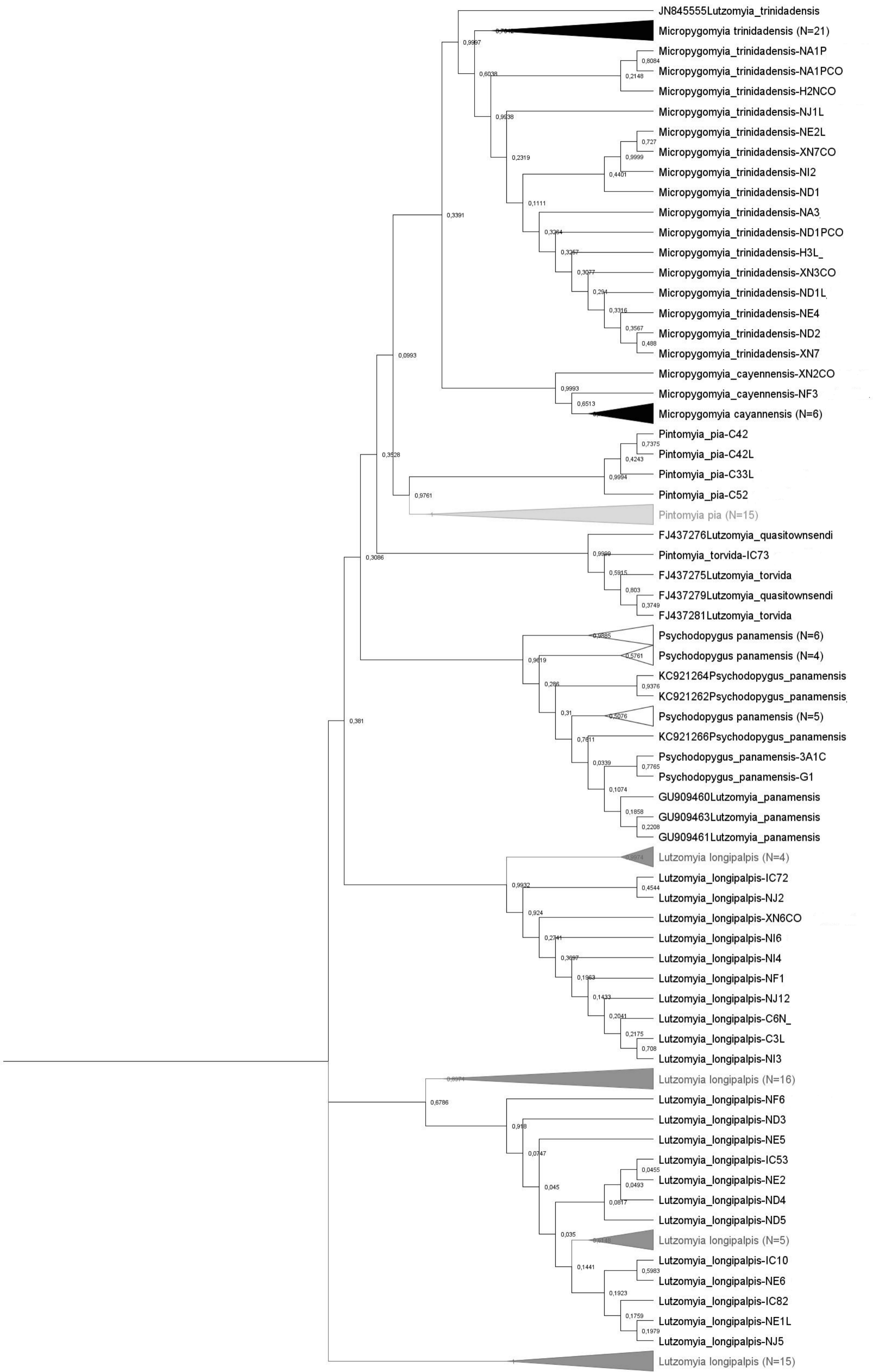

Supplement: Supplementary file 5 — Supplementary Figure S3. [file 41598_2021_85763_MOESM5_ESM.pdf]
